# Age of acquisition and allophony in Spanish-English bilinguals

**DOI:** 10.3389/fpsyg.2014.00288

**Published:** 2014-04-21

**Authors:** Jessica A. Barlow

**Affiliations:** Phonological Typologies Lab, School of Speech, Language, and Hearing Sciences, San Diego State UniversitySan Diego, CA, USA

**Keywords:** bilingual, adult, allophones, acoustic, lateral approximant

## Abstract

This study examines age of acquisition (AoA) in Spanish-English bilinguals’ phonetic and phonological knowledge of /l/ in English and Spanish. In English, the lateral approximant /l/ varies in darkness by context [based on the second formant (F2) and the difference between F2 and the first formant (F1)], but the Spanish /l/ does not. Further, English /l/ is overall darker than Spanish /l/. Thirty-eight college-aged adults participated: 11 Early Spanish-English bilinguals who learned English before the age of 5 years, 14 Late Spanish-English bilinguals who learned English after the age of 6 years, and 13 English monolinguals. Participants’ /l/ productions were acoustically analyzed by language and context. The results revealed a Spanish-to-English phonetic influence on /l/ productions for both Early and Late bilinguals, as well as an English-to-Spanish phonological influence on the patterning of /l/ for the Late Bilinguals. These findings are discussed in terms of the Speech Learning Model and the effect of AoA on the interaction between a bilingual speaker’s two languages.

## INTRODUCTION

It is widely established that a bilingual’s two languages interact; this interaction happens during the acquisition process for both children and adults, and continues after the languages have been mastered with native-like competence (Paradis, 2001a, b; Cook, 2003; Flege et al., 2003; Fabiano and Goldstein, 2005; Flege, 2007; Barlow et al., 2013). Such interaction has been described at numerous levels of linguistic structure, from pragmatic to syntactic to lexical to phonological (e.g., Pavlenko and Jarvis, 2002; Cook, 2003; Dussias, 2003; Flege et al., 2003; Dussias and Sagarra, 2007; Flege, 2007; Amengual, 2012). At the phonological level, interaction has been reported for prosodic and other suprasegmental structure, segmental patterns, and even subsegmental patterns that pertain to allophonic and other acoustic-phonetic phenomena (Paradis, 2001a, b; Lleó et al., 2003; Kehoe et al., 2004; Mennen, 2004; Fabiano and Goldstein, 2005; Lleó, 2006; Fabiano-Smith and Barlow, 2010; Barlow et al., 2013).

The challenge for researchers is to be able to predict if, where, and how interaction will occur. As far as speaker extrinsic factors are concerned, frequency, markedness, and similarity play a role (Lleó et al., 2003; Broselow, 2004; Fabiano-Smith and Goldstein, 2010). For instance, interaction is more likely to occur on properties that are similar or shared between two languages than those that are dissimilar or unshared (e.g., Flege, 1995, 2007; Flege et al., 1999, 2003; MacWhinney, 2004). Speaker intrinsic factors are also relevant to predicting interaction between a bilingual’s two languages. Specifically, the nature and extent of interaction depends on the age of the speaker, the age at which each language was acquired, the amount of input and output in both languages, and the level of proficiency and dominance in the two languages (e.g., Flege, 1991, 2002; Flege et al., 1995, 1997, 1999, 2002; Thornburgh and Ryalls, 1998; Guion, 2003; Fowler et al., 2008; Simonet, 2010; Antoniou et al., 2011; Lee and Iverson, 2012).

In this study, we focus on Spanish-English bilinguals who represent a significant and growing population in the US, particularly Southern California (US Census Bureau, 2004). There exists a large body of research on the speech of Spanish-English bilinguals, with both child and adult populations. Allophonic variation in this language group has also been of particular interest for researchers, many of whom have focused on voice onset time (VOT) for word-initial (WI) plosive stops (e.g., Flege and Eefting, 1987; Flege, 1991; Yavaş, 1996; Thornburgh and Ryalls, 1998; Amengual, 2012; López, 2012). VOT is relevant because English has an allophonic rule that governs the distribution of long- and short-lag voiceless stops, with long-lag stops occurring word-initially, whereas Spanish has only short-lag voiceless stops. Other studies of allophonic phenomena in Spanish-English bilinguals have considered the distribution of voiced stops and spirants (Zampini, 1994; e.g., Zampini, 1996; Eckman and Iverson, 1997; Barlow, 2003). In this case, Spanish has the allophonic rule governing the distribution of the two types of sounds, whereas English does not exhibit such alternation. Not surprisingly, these prior studies have demonstrated that bilinguals show interaction between their two languages in terms of the allophonic patterns evaluated, though results vary due to factors such as age of acquisition (AoA) and dominance, as described above.

Few studies have focused on other allophonic phenomena in evaluation of interaction between Spanish-English bilinguals’ two languages. Thus, to expand on our understanding of Spanish-English bilinguals’ productions of allophonic phenomena, the current study focuses on an allophonic pattern of English that has received relatively little attention in prior research on bilinguals: the distribution of /l/ allophones in English. The lateral approximant /l/ is of interest here because it is a phoneme that is shared between the two languages, but is produced differently, both in terms of its acoustic-phonetic properties and its allophonic distribution.

Specifically, Spanish /l/ is typically described as “clear,” in that it is perceived as more consonantal in quality, regardless of context, whereas American English /l/ is characterized as relatively “dark” in all contexts, in that it is perceived as more vowel-like in quality (Wells, 1982a, b; Huffman, 1997; Whitley, 2002; Recasens, 2004, 2012; Recasens and Espinosa, 2005). Using x-ray microbeam technology, Sproat and Fujimura (1993) determined that this darker quality for American English /l/ is attributed to two co-occurring gestures: a consonantal tongue-tip gesture and a vocalic tongue-dorsum gesture. Spanish /l/ is assumed to lack this latter vocalic gesture, given its perceptually “clearer” quality.

Furthermore, American English has an allophonic velarization rule that Spanish lacks. This rule governs the distribution of [ɫ], which occurs in the syllable rhyme (e.g., “meal” [miɫ], “milk” [mıɫk], and “candle” [kændɫ]), and [l], which occurs in the syllable onset (e.g., “lease” [lis], “ply” [plaı]). The [ɫ] is perceived as even darker than onset [l] in American English^1^, and this, per Sproat and Fujimura (1993), is due to the relative timing of the aforementioned consonantal and vocalic gestures. Specifically, for onset [l], the consonantal gesture precedes or occurs simultaneously with the vocalic gesture; for rhymal [ɫ], the vocalic gesture precedes the consonantal gesture (see also Browman and Goldstein, 1992; Gick, 2000, 2003). The different sequencing of these gestures is associated with syllable position: consonantal gestures tend to occur on the periphery (margins) of syllables, while vocalic gestures occur closer to the peak (rhyme) (Sproat and Fujimura, 1993; Huffman, 1997; Gick, 2000, 2003). In contrast to English, the Spanish /l/ phoneme does not vary by context; thus, the consonantal gesture associated with Spanish /l/ is assumed to be relatively consistent across contexts, though, per Gick et al. (2006), some degree of dorsal constriction in postvocalic contexts is predicted to occur.

Note that the relative timing of consonant and vocalic gestures associated with American English /l/ has been described as occurring along a continuum that is dependent on proximity to the syllable margins and peaks (Sproat and Fujimura, 1993; Gick, 2000, 2003; Gick et al., 2006). Morphological and prosodic factors also have been noted to affect the relative darkness of American English /l/ (Hayes, 2000; Oxley et al., 2007). However, for the purposes of the current study, which evaluates only two contexts – WI onset singletons and word-final (WF) coda singletons – a categorical distinction between articulations for the two /l/ allophones is assumed (see also Yuan and Liberman, 2011, for an argument in support of this categorical distinction).

These clear and dark /l/ varieties manifest acoustically via differing resonant frequencies. Clear /l/ has a high second formant (F2) value and a large difference between F2 and the first formant (F1) (Recasens, 2004; Recasens and Espinosa, 2005; Yuan and Liberman, 2009, 2011; Proctor, 2010; Simonet, 2010). Dark /l/, in contrast, is associated with lower F2 values and a smaller F2-F1 difference.

As stated above, the Spanish /l/ is described as clear in all word positions: F2 shows only subtle change by context, sometimes with slightly *higher* values in WF position. For instance, Quilis et al. (1979) reported F2 values of 1800 Hz for /l/ in the syllable [li], but 1960 Hz for the syllable [il] in adult male speakers of Castilian Spanish. Similarly, F2 values for /l/ were reported at 1400 Hz for [lu], but 1410 Hz for [ul]. In contrast, due to the allophonic velarization rule, F2 varies more substantially by context in American English, with much lower values word-finally than word-initially. For example, Lehiste (1964) reported F2 values of 1185 Hz for /l/ in [li] for adult male speakers of American English, but 740 Hz for [iɫ]. Similarly, F2 values for /l/ were reported to be 1070 Hz for [lu], but 655 Hz for [uɫ]. Collapsing across vowel contexts, these studies show an average adult male F2 value of 1587 Hz word-initially and 1630 Hz word-finally in Spanish, and 1052 Hz word-initially and 755 Hz word-finally in English. Scale factors for adult females are estimated to be 1.17–1.19 (Peterson and Barney, 1952; Hillenbrand et al., 1995; Chládková et al., 2011). Based on this, estimates of female Spanish F2 values (using the scale factor of 1.18) would be around 1873 Hz word-initially and 1923 Hz word-finally, and English values would be at around 1241 Hz word-initially and 891 Hz word-finally. Averaging across the sexes, Spanish F2 values are estimated to be 1730 Hz word-initially and 1777 Hz word-finally; English F2 values are estimated to be 1147 Hz word-initially and 823 Hz word-finally.

Prior research suggests that these phonetic and phonological differences associated with /l/ are likely areas for interaction between Spanish-English bilinguals’ two languages. For instance, in a recent acoustic study of Spanish-English sequential bilingual children’s productions of /l/ in both languages (Barlow et al., 2013), it was determined that even young children were distinguishing their /l/ productions by language and context, such that they produced Spanish /l/ with monolingual-like F2 and F2-F1 values that were overall higher than those for English /l/. They also demonstrated knowledge of the English velarization pattern, in that they produced a syllable-final /l/ with lower, monolingual-like F2 and F2-F1 values as compared to /l/ produced in onset contexts. One interesting finding that emerged, however, was that the bilingual children produced onset /l/ in Spanish and English with similar F2 and F2-F1 values. In both their languages, they produced a very clear /l/ in that context, with average F2 values above 1800 Hz. Thus, while they appeared to produce a distinction between Spanish and English /l/ in the context in which the velarization rule applied (in the syllable rhyme), they did not produce a distinction between Spanish and English /l/ in the onset.

These findings can be interpreted in terms of Flege’s Speech Learning Model (SLM; Flege, 1995, 2007). According to the SLM, a bilingual’s two linguistic systems share a “common phonological space.” This shared space can cause bidirectional interaction to occur throughout the lifespan, regardless of the age at which the second language (L2) was acquired or the number of years speaking that L2, though the extent, type, and direction of interaction will be influenced by these factors as well as other factors described above, such as dominance and use (Flege, 2002, 2007; Flege and MacKay, 2004). Per the SLM, interaction may occur via perceptual assimilation, where a contextual allophone of the L2 is perceived as phonetically equivalent to an existing phonetic category in the first language (L1), causing the two categories to be merged into one that reflects the properties of both languages (the “Merger Hypothesis”; Flege, 1987, 1995). The more similar two phonetic categories are in the L1 and L2, the more likely such category assimilation is to occur. In this case, productions of the contextual allophones for the L1 and the L2 may occur as intermediate to those of monolinguals in the respective languages. Alternatively, interaction may also occur via perceptual dissimilation, where a new phonetic category of the L2 is created, but, due to its similarity to an already-existing category in the L1, the two categories dissimilate from each other to maintain the contrast and prevent “crowding” of the shared phonological space. In this case, productions of the contextual allophones for the L1 and the L2 may be more distinct (that is, exaggerated) as compared to those of monolinguals of the respective languages (Flege, 1987, 1995).

Applying the SLM to the findings from the study of Spanish-English bilingual children’s productions of /l/ (Barlow et al., 2013), it can be assumed that the children classified the syllable-initial /l/ phones of Spanish (the L1) and English (the L2) as phonetically equivalent, and therefore merged those two categories, which resulted in the acoustically similar productions in that context. It is also assumed that the children classified the syllable-final /l/ phones of each language as more distinct from one another (as compared to the syllable-initial phones), and established separate phonetic categories for them, which resulted in the acoustically distinct productions in that context for the two languages.

This raises the question of whether these children would continue to produce the same /l/ in Spanish and English in adulthood, or if their English onset /l/ productions would gradually become more monolingual-like with added input from the surrounding linguistic community (Flege, 1987; Barlow et al., 2013). On the one hand, as children, they were still in the process of learning both languages, and fine-tuning of articulatory and acoustic properties of speech continues well into adolescence (Kent, 1976; Walsh and Smith, 2002; Oh, 2005; Vorperian et al., 2009). Thus, as they reach adulthood, the children’s /l/ productions might become more monolingual-like in both languages.

Yet, there may be no motivation on the children’s part to change how they articulate /l/. The use of a Spanish-like clear /l/ in English, even in syllable-final contexts where velarization would typically apply, is not likely to compromise understanding or perhaps even detection of an accent (Port and Mitleb, 1983; Flege and Eefting, 1987; MacKay et al., 2001; Flege, 2002, 2007). Indeed, clear /l/ is an acceptable variant in English, and dialectal and idiolectal variation in /l/ darkness does occur among (monolingual) native English speakers (Wells, 1982a, b; Frazer, 1996; Hayes, 2000; Hawkins and Nguyen, 2004; Carter and Local, 2007; Ladefoged and Johnson, 2011). This is unlike differences in VOT, for example, where a breakdown in communication could occur at worst, or an accent would be noticeable at best. For instance, in English, categorically voiced stops are produced with VOTs that are similar in duration to categorically voiceless stops in Spanish. Thus, if a Spanish learner of English were to pronounce the English word “park” with a short-lag Spanish VOT, instead of the long-lag VOT (aspiration) that is typical of a native English speaker, this might be perceived by native English speakers as “bark.” Given that no such phonemic overlap occurs for the /l/ allophones, it stands to reason that Spanish-English bilinguals might be more likely to maintain this merged phonetic category for /l/ in the two languages, via assimilation.

As mentioned above, the age at which bilinguals acquire their two languages plays an important role in the extent and type of interaction that can occur between their languages. Converging evidence suggests that there may be multiple critical (or sensitive) periods for acquisition of different domains of language. Accordingly, there is a higher (older) upper age limit for acquisition of morphosyntax and semantics, which is around 16 years of age, as compared to that for phonology, which is around the age of 5 years (Flege et al., 1999; Scovel, 2000; Newport et al., 2001). The L1 effects on the L2 are more likely to be observed if L2 acquisition occurs after these cutoff ages. Nevertheless, fine-tuning of articulatory aspects of the sound system continues into adolescence, as mentioned above, as does phonemic category formation (Hazan and Barrett, 2000).

Whether phonetic category assimilation or dissimilation occurs in L2 acquisition is also dependent on whether L1 category formation has occurred, and the extent to which it has occurred (Hazan and Barrett, 2000; Flege, 2007). If category formation has already occurred, or has developed well in advance of that for the L2, then the L2 learner is more likely to exhibit category assimilation, because the L1 categories serve as “strong attractors” for phonetically similar sounds in the L2 (Flege and MacKay, 2011). In contrast, if L1 category formation has not yet occurred, or is not far in advance of that of the L2, then the learner is more likely to establish separate phonetic categories for the two languages. In turn, dissimilation is more likely to occur, motivated by the avoidance of a crowded phonological space (Flege, 1995, 2002; Flege and MacKay, 2011).

Related to this is the observation that the later the AoA of the L2, the greater the use of the L1; conversely, the earlier the AoA of the L2, the greater the use of the L2 (Flege et al., 1997; Flege and MacKay, 2011). Further, studies of perception and production that manipulate the factors of AoA and L1 use have indicated that early bilinguals who exhibit low L1 use are the most likely to establish a new L2 category, as evidenced by exaggerated differentiation of the phonetically similar sounds (Flege et al., 1997, 2003; Flege and MacKay, 2004).

The purpose of the current study was to add to our understanding of Spanish-English bilinguals’ knowledge and use of /l/ in their two languages, by considering the productions of adult bilinguals who acquired Spanish from birth and acquired English either simultaneously or sometime before adulthood. Further, because AoA is known to impact the extent of interaction between a bilingual’s two languages (Flege, 1991; Flege et al., 1995, 1999; MacLeod and Stoel-Gammon, 2010), a second goal was to determine if – when dominance and extent of language use were held constant – the age at which bilinguals learned English impacted their productions of /l/ in their two languages.

Toward this end, we acoustically analyzed /l/ productions of Early Bilinguals, Late Bilinguals, and English Monolinguals in English and Spanish in terms of their relative darkness as indicated by F2 and F2-F1 measurements in onset (WI) and rhyme (WF) contexts. It was predicted that Early and Late Bilinguals would have phonological systems that are comparable to those of English Monolinguals with respect to the patterning of /l/. That is, they would show knowledge of the allophonic velarization rule in English by producing a darker /l/ word-finally as compared to initially. Given that sequential bilingual children evidence knowledge of this phonological pattern (Barlow et al., 2013), it stands to reason that adult bilinguals who acquired both languages before adulthood would as well.

Similarly, it was predicted that Early and Late Bilinguals would have phonological systems that are comparable to those of Spanish monolinguals (as described in prior research) with respect to the patterning of /l/. That is, they would show little to no difference in their /l/ productions in word-initially vs. finally. Once again, given the prior evidence that sequential bilingual children show knowledge of how Spanish /l/ patterns (Barlow et al., 2013), it stands to reason that the adult bilinguals would do so as well.

It was also predicted that Early and Late Bilinguals would produce a phonetic distinction between their Spanish and English /l/ sounds. That is, independent of the allophonic velarization rule in English, the bilinguals would produce a clearer /l/ in Spanish than in English. However, it was predicted that the Late Bilinguals, whose Spanish phonetic categories for /l/ were further developed when they began acquiring English, would produce English and Spanish /l/s in both contexts that were intermediate in clearness to those of monolinguals in both languages, which would be indicative of category assimilation (Flege, 1995, 2002). In contrast, the Early Bilinguals, who were still in the process of forming Spanish phonetic categories for /l/ when acquiring English, were predicted to produce /l/s in both languages as distinct from one another in each context, which would be indicative of category formation for both languages (Flege, 1995, 2002). Dissimilation was not predicted to occur for the Early Bilinguals, because the /l/ variants in the two languages do not overlap with other existing phonetic categories within either language, as discussed above.

## MATERIALS AND METHODS

### PARTICIPANTS

Thirty-eight college students participated in the study. This included 11 Early Spanish-English Bilinguals with a mean age of 20.6 years (SD = 1.8 years), seven of whom were female; 14 Late Spanish-English Bilinguals with a mean age of 20.4 years (SD = 1.2 years), 12 of whom were female; and 13 English Monolinguals with a mean age of 20.5 years (SD = 1.6 years), eight of whom were female. The groups did not differ significantly in terms of age, *F*(2,35) = 0.05, *p* = 0.95.

Eligible participants were required to have normal or corrected-to-normal vision, normal hearing and oro-motor function, as well as no history of developmental, cognitive, speech, or language difficulties. These restrictions were necessary for completion of the tasks associated with the study and for controlling for interspeaker differences as much as possible.

All participants completed a detailed questionnaire regarding their language background, use, and proficiency (adapted from Gutiérrez-Clellen and Kreiter, 2003). They answered specific questions regarding languages and dialects spoken, where they grew up, specific regions of the US and Mexico in which they resided, the age(s) at which they learned their language(s), and how many hours per day they used each language and with whom and in what context. In order to control for language and dialect effects, only participants who spoke varieties of Spanish and/or English from the Southern California (US) and Baja California (Mexico) region were included in the study.

Participants also self-rated their receptive and expressive abilities in English and Spanish on a scale from “0” (unable to understand/speak the language) to “4” (native-like ability to understand/speak the language). Participants were classified as Spanish-English bilingual if they rated themselves at 3 or 4 for receptive and expressive abilities in both English and Spanish. Participants were classified as English monolingual if they reported that they knew English from birth, they rated themselves with a 3 or 4 for English and a 0 for Spanish, and they did not report knowledge of any other spoken language. Chi-square tests revealed no significant differences between the three participant groups in terms of ratings for receptive and expressive abilities for English, or between the Early and Late Bilinguals in terms of receptive and expressive abilities for Spanish (all *p*s > 0.05).

Based on their responses on the questionnaire, participants were characterized as “Early Bilinguals” if they learned Spanish from birth and acquired English before the age of 5 years, or as “Late Bilinguals” if they learned Spanish from birth and acquired English after the age of 6 years. This criterion was determined *a priori*, and was based on prior research findings that show the age of 5 years as the upper limit for the critical period for native- or monolingual-like phonological acquisition, as described above (McLaughlin, 1978; Flege, 1991; Flege et al., 1995; Hamers and Blanc, 2000; Scovel, 2000; Genesee et al., 2004; Gildersleeve-Neumann and Wright, 2010; Lee and Iverson, 2012)^2^. The Early Bilinguals had a mean AoA of English of 2.4 years (SD = 1.7 years), and the Late Bilinguals had a mean AoA of 8.3 years (SD = 2.0 years). This difference in AoA between the two groups was significant, *t*(23) = -7.71, *p* < 0.001.

Responses to the portion of the questionnaire that addressed language speaking and listening contexts were quantified in order to compare participants in terms of amount of input and output in their language(s). A “1” was scored for use of English only in a given speaking/listening context, and a “5” was scored for use of Spanish only. The scores were then averaged across the listening (input) and speaking (output) contexts. Thus, a score closer to 1 would reflect greater input/output in English, while a score closer to 5 would reflect greater input/output in Spanish. In contrast, a score of 3 would reflect a more or less balanced bilingual in so far as language input/output was concerned. Not surprisingly, the English monolinguals’ scores were 1.04 (SE = 0.04) for input and 1.03 (SE = 0.03) for output^3^. The Early Bilinguals’ mean input was 2.38 (SE = 0.24) and their mean output was 2.41 (SE = 0.27). The Late Bilinguals’ mean input was 2.51 (SE = 0.20) and their mean output was 2.56 (SE = 0.21). Analyses of variance (ANOVAs) showed an effect of background for input, *F*(2,35) = 21.8, and for output, *F*(2,35) = 19.9, *p* < 0.001. *Post hoc* tests using Bonferroni correction revealed that, for both input and output, the monolinguals had significantly lower scores than did the Early and Late Bilinguals (*p* < 0.001), consistent with their monolingual status. The Early and Late Bilinguals did not significantly differ from one another on any single speaking or listening context, or on their overall input or output scores (*p* = 1.00). The scores indicate that both groups of bilinguals showed slightly greater input/output for English, which is not surprising given that they are students at a predominately English-speaking university. Nevertheless, both groups maintain high usage of Spanish as well, which is supported by the Southern California community which boasts a large Spanish-speaking population (US Census Bureau, 2003).

### STIMULI AND RECORDING PROCEDURES

To evaluate the participants’ /l/ productions by language and context, a 177-item list was created for each language using 59 words sampled three times each in random order. These words were similar in phonetic form across the two languages and balanced for adjacent vowels. Of these 59 words, there were five mono- or di-syllabic WI /l/ words and five mono- or di-syllabic WF /l/ words for each language. An additional five intervocalic /l/ words and five WI plosive + /l/ cluster words were also included, but are not analyzed herein^4^. The remaining words included voiceless stop consonants, which were targeted as part of a separate study. Of relevance to the current study were the 10 /l/ words for each language, sampled three times each per participant, yielding 30 attempts per participant per language. Refer to **Table 1** for a list of the stimuli analyzed in this study and their corresponding phonetic representations based on the Southern California and Baja California dialects. Broad transcription is used except in the case of the representation of the /l/ phoneme (Hualde, 2005).

**Table 1:** English and Spanish stimuli.

English	Spanish
**WI**
lease [lis]	*liso* [liso] “flat, smooth”
lay [leı]	*ley* [lei] “law”
loss [las]	*laso* [laso] “weary”
load [lo℧d]	*lodo* [lodo] “mud”
loose [lus]	*luz* [lus] “light”
**WF**
meal [miɫ]	*mil* [mil] “thousand”
mail [meıɫ]	*miel* [miel] “honey”
mall [maɫ]	*mal* [mal] “bad”
soul [soɫ]	*sol* [sol] “sun”
tool [tuɫ]	*tul* [tul] “tulle”

Participants were seated in a quiet room, with a SONY electret condenser MS907 microphone placed approximately 6 inches in front of them. All utterances were digitally recorded onto an Edirol R-09HR MP3 digital recorder. Participants were asked to read each word from the 177-item list in a carrier phrase [“Say again,” or *Di __ ahora* (“Say __ now”)]. To control for order effects and to aid in separation of language modes for the bilinguals, the Spanish and English tasks were presented in random order across participants and were separated in time by completion of the language questionnaire described above. The digital recording files were then transferred to a computer for the purpose of acoustic analysis.

As stated, there were 30 target /l/ productions per participant per language, yielding a total of 1140 productions in English (for the 38 participants from all three groups) and 750 productions in Spanish (for the 25 participants from the two bilingual groups). Some items were excluded due to extraneous noise in the signal (e.g., the participant bumped against the table) or because the participant mispronounced the target word (e.g., “load” pronounced as [laυd]). With these forms excluded, the number of items analyzed was reduced to 1135 words in English and 747 words in Spanish.

### ANALYSES

In order to compare productions of /l/ by language and context, formant measurements were required in order to compare F2 values and the F2-F1 differences. To do this, the digital recordings were acoustically analyzed with Praat software (v. 5.0.26; Boersma and Weenink, 2008) by student research assistants who were trained extensively in the use of the software and in the analysis of formants of vowels and approximants. To make the formant measurements, the midpoint of each /l/ production was identified both visually (from waveform and spectrogram displays) and perceptually (via headphones). Then, the mouse cursor on the computer was placed at the identified midpoint of each target /l/ production and the “Get Formants” command was used to obtain values for F1 and F2. From these values the raw F2-F1 difference was calculated. F2 values for the vowels /i/ and /o/ in the above word list were also determined for the purpose of normalization (as described below) using the same procedure within Praat.

Because this method of acoustic analysis is somewhat subjective, interjudge reliability was calculated in the following two ways. First, correlation analyses were conducted for 15% of the stimuli analyzed. Correlation between the F1 measures for the two judges for 283 items was *r*(281) = 0.777, *p* < 0.001. Correlation between the F2 measures was *r*(281) = 0.894, *p* < 0.001. Second, the absolute difference in measures was determined (following Shriberg et al., 1997). This is calculated by finding the mean of the first judge’s measures, calculating the mean of the absolute value of the difference between the first judge’s mean and that of the second judge’s measures, and then dividing the absolute mean by the original mean. Based on the same 283 items, the mean absolute difference for F1 and F2 was 10 and 8%, respectively.

The raw F2 and F2-F1 values were averaged across all productions per context for each speaker. To account for individual and sex-based differences attributed to vocal tract size and length, all raw F2 measures were normalized using the S-procedure of Watt and Fabricius (2002), following Simonet (2010). Comparisons of vowel normalization methods (Adank et al., 2004; Flynn and Foulkes, 2011) indicate that, for conducting language variation research such as with the present study, normalization procedures that are category extrinsic, formant intrinsic, and speaker intrinsic are best for reducing interspeaker variation that is attributed to anatomical and physiological differences and for preserving interspeaker variation that is attributed to language and dialect differences (Flynn and Foulkes, 2011). Because the focus of the current study was on the lateral approximant, the vowel sounds /i/ and /o/ were taken into consideration in the normalization procedure, thereby making the process category extrinsic. These two vowels were selected to represent the front and back extremes of the vowel space for the purposes of this study. The vowel /o/ was selected instead of /u/, which in the California English dialect is known to be fronter in the vowel space than is /o/ (Hagiwara, 1995, 1997; Labov et al., 2006; Grijalva et al., 2013)^5^. In the Spanish spoken in this area, /o/ and /u/ have similar degrees of backness (Grijalva et al., 2013). The normalization procedure also included the lateral approximant formant values (formant intrinsic) for each speaker (speaker intrinsic). The S-procedure of Watt and Fabricius (2002) as described by Simonet (2010) and by Flynn and Foulkes (2011) involves determining the centroid F2 value for a given speaker’s vowel space. To do this, the mean F2 values for the /i/ vowel and the /o/ vowel were obtained (based on the productions in the word list discussed above); then, a grand mean of those two vowels was determined which served as the centroid F2 value (Simonet, 2010). The normalized F2 value for each /l/ production was determined by dividing the raw F2 value by the centroid F2 value.

Separate repeated measures ANOVAs with follow-up analyses using the Bonferroni procedure compared mean normalized F2 values and raw F2-F1 difference values across all /l/ tokens by background (Monolingual vs. Early vs. Late Bilingual), and context (WI vs. WF) for each language (English and Spanish). Additional separate repeated ANOVAs also were conducted for within-group comparisons of Spanish and English productions for the Early and Late Bilinguals.

## RESULTS

The means and standard errors of the means for the raw F1 and F2 values of /l/ productions are shown in **Table 2**, organized by language, background, and context. Compared to monolingual data reported in prior research (as discussed in the Introduction), the raw F2 values are notably high across groups, closer to the estimated female values for each language, and this is attributed to the fact that the current study includes more females than males (Peterson and Barney, 1952; Hillenbrand et al., 1995). The one exception is WF position in Spanish, which for both Early and Late Bilinguals is lower than what is reported in prior studies.

**Table 2:** Means (and standard errors) of raw F1 and F2 values for English and Spanish.

	F1		F2	
	WI	WF	WI	WF
**English**
Late Bilinguals	407 (21)	520 (28)	1508 (64)	1161 (46)
Early Bilinguals	403 (24)	509 (23)	1420 (77)	1123 (30)
Monolinguals	391 (12)	505 (16)	1235 (48)	1055 (32)
**Spanish**
Late Bilinguals	385 (20)	399 (17)	1838 (43)	1698 (47)
Early Bilinguals	386 (19)	405 (10)	1716 (41)	1644 (51)

The following sections present the results of the between-group comparisons for English and Spanish as well as the within-group comparisons for the Early and Late Bilinguals for normalized F2 and raw F2-F1 differences.

### ENGLISH

First, a between-groups analysis compared English /l/s produced by the Monolinguals and the Early and Late Bilinguals in order to test the prediction that bilinguals have an English phonological system comparable to that of monolinguals. Support for this would be evident from the occurrence of higher mean normalized F2 and raw F2-F1 values in WI as compared to WF contexts, as predicted by the allophonic velarization rule in English.

**FIGURE 1:**
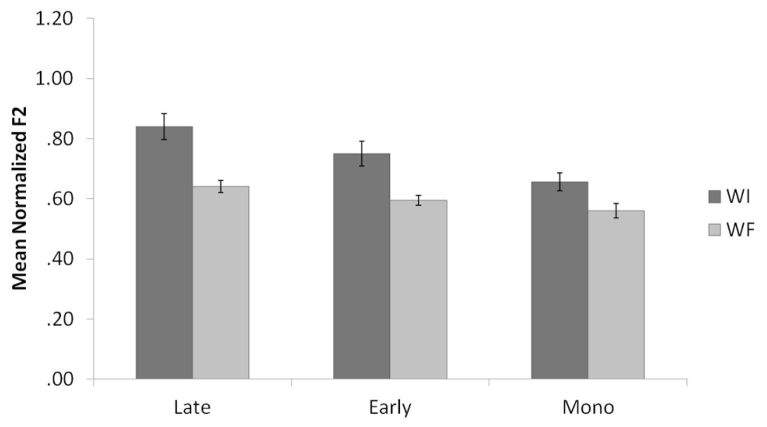
**Mean normalized F2 values by context for Late Bilinguals, Early Bilinguals, and Monolinguals for English.** Error bars refer to standard errors; WI refers to word-initial, and WF refers to word-final.

**Figure 1** shows the means and standard errors for the normalized F2 values of English /l/ productions, organized by background and context. Results of the English normalized F2 analysis revealed a main effect for context, *F*(1,35) = 47.3, *p* < 0.001. WI /l/s had significantly higher F2 values than WF /l/s, consistent with the allophonic patterning of /l/ in English. There was also a main effect for background, *F*(2,35) = 7.5, *p* = 0.002. Pairwise comparisons revealed that the Late Bilinguals produced /l/ with significantly higher F2 values compared to the Monolinguals. The Early Bilinguals did not significantly differ from the Late Bilinguals or the Monolinguals. Moreover, there was no significant interaction between context and background (*p* = 0.15).

**FIGURE 2:**
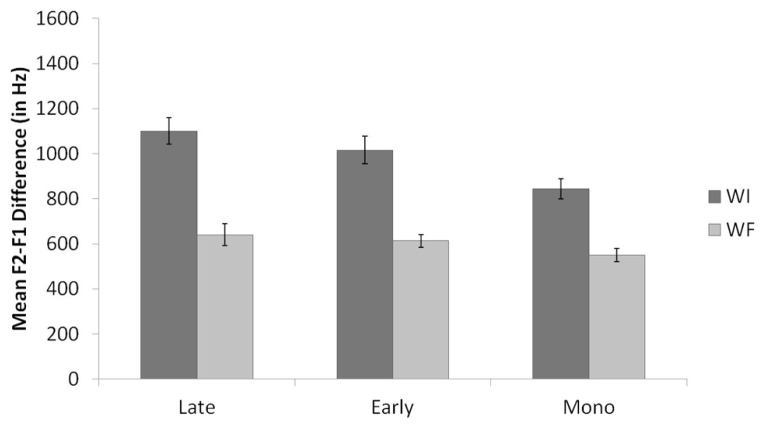
**Mean raw F2-F1 values by context for Late Bilinguals, Early Bilinguals, and Monolinguals for English.** Error bars refer to standard errors.

**Figure 2** displays the means and standard errors for the raw F2-F1 differences for English /l/ productions, organized by background and context. Similar to the results of the normalized F2 analysis, results of the English F2-F1 analysis revealed a main effect for context, *F*(1,35) = 102.0, *p* < 0.001. WI /l/s had significantly higher F2-F1 values than WF /l/s, consistent with the allophonic patterning of /l/ in English. There was also a main effect for background, *F*(2,35) = 6.8, *p* = 0.003. Pairwise comparisons revealed that the Late Bilinguals produced /l/ with significantly higher F2 values compared to the Monolinguals (*p* = 0.003). The difference between Early Bilinguals and Monolinguals approached significance (*p* = 0.08), with the Early Bilinguals producing higher F2-F1 values than the Monolinguals. The Early Bilinguals and Late Bilinguals did not differ significantly from each other. Additionally, the interaction between context and background was not significant (*p* = 0.19).

Thus, the results support the prediction: both groups of bilinguals demonstrated phonological knowledge of the allophonic velarization rule in English by producing a lower normalized F2 and a smaller F2-F1 difference in WF as compared to WI position. However, the findings also illustrate that the Late Bilinguals differ from the Monolinguals given their production of overall higher F2 and F2-F1 values. These higher F2 and F2-F1 values are assumed to be due to interference from the Late Bilinguals’ knowledge of Spanish, which has a clearer /l/.

### SPANISH

Next, a between-groups analysis evaluated the bilinguals’ Spanish /l/ productions in order to test the prediction that Early and Late Bilinguals would have phonological systems that are comparable to those of Spanish monolinguals with respect to the patterning of /l/ (as described in prior research). Support for this would be evidenced by a lack of difference by context for normalized F2 and raw F2-F1 values for both groups.

**FIGURE 3:**
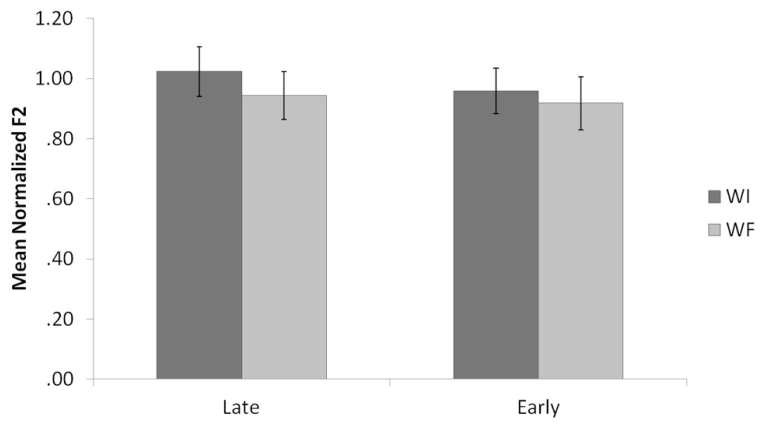
**Mean normalized F2 values by context for Late Bilinguals and Early Bilinguals for Spanish.** Error bars refer to standard errors.

**Figure 3** shows the means and standard errors for the normalized F2 values of Spanish /l/ productions, organized by background and context. Results of the Spanish normalized F2 analysis revealed a main effect for context, *F*(1,23) = 20.5, *p* < 0.001. WI /l/s had significantly higher F2 values than WF /l/s, which was unexpected, given that prior research reports WFs /l/s that are similar to or slightly higher than WI /l/s in Spanish. There was no main effect for background (*p* = 0.15), nor was there a significant interaction between context and background (*p* = 0.18).

**FIGURE 4:**
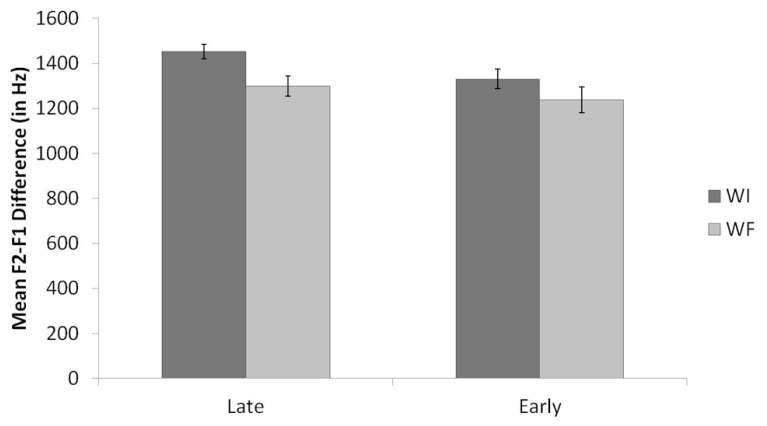
**Mean raw F2-F1 values by context for Late Bilinguals and Early Bilinguals for Spanish.** Error bars refer to standard errors.

**Figure 4** shows the means and standard errors for the raw F2-F1 differences for Spanish /l/ productions, organized by background and context. Results of the Spanish F2-F1 analysis also revealed a main effect for context, *F*(1,23) = 14.7, *p* = 0.001. Once again, WI /l/s had significantly higher F2-F1 values than WF /l/s. The main effect of background approached significance (*p* = 0.10), with Late Bilinguals producing higher F2-F1 values than Early Bilinguals. There was no significant interaction between context and background (*p* = 0.34).

These findings go against the original prediction. The effect of context indicates that, generally speaking, the Bilinguals in this study in fact produced a difference by context for /l/ in Spanish by producing a darker /l/ in WF position, which is inconsistent with prior descriptions of the Spanish language (see Introduction), and is suggestive of an effect of the English phonological system on the Spanish. However, the marginally significant effect of background for F2-F1 values suggests that Late Bilinguals may in fact be driving this contextual pattern. To further evaluate this possibility, we consider our within-group comparisons next.

### BILINGUALS: ENGLISH vs. SPANISH

Separate repeated measures ANOVAs were also completed to make within-group comparisons by language and context for the Early and Late Bilinguals, respectively. This tested the prediction that Early and Late Bilinguals would produce a phonetic distinction between their English and Spanish /l/ phonemes, and allowed for further determination of whether Early and Late Bilinguals showed different profiles with respect to this phonetic distinction.

**FIGURE 5:**
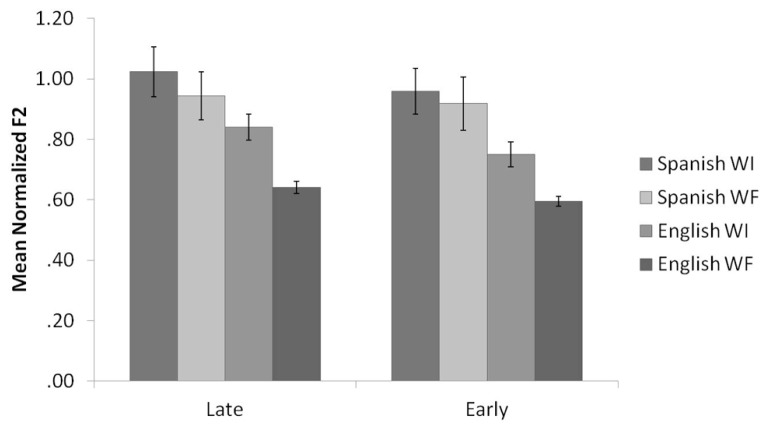
**Mean normalized F2 values by language and context for Late Bilinguals and Early Bilinguals.** Error bars refer to standard errors.

**Figure 5** displays the means and standard errors for the normalized F2 values of Spanish and English /l/ productions once again, organized by language, background, and context. Results of the normalized F2 analyses revealed, not surprisingly, a significant difference across the four measures for Late Bilinguals, *F*(3,39) = 37.4, *p* < 0.001, and for Early Bilinguals, *F*(3,30) = 58.7, *p* < 0.001.

*Post hoc* tests using Bonferroni correction for normalized F2 values for the Late Bilinguals revealed that Spanish WI /l/s were significantly higher than Spanish WF, English WI, and English WF /l/s. In addition, Spanish WF /l/s were significantly higher than English WF /l/s, and English WI /l/s were significantly higher than English WF /l/s. (All *p*s < 0.01.) Spanish WF and English WI /l/s were not significantly different (*p* = 0.39).

*Post hoc* tests for the Early Bilinguals revealed that Spanish WI and WF /l/s were significantly higher than English WI and WF /l/s. Moreover, English WI /l/s were higher than English WF /l/s. (All *p*s < 0.01.) Spanish WI and WF /l/s were not significantly different (*p* = 0.42).

**FIGURE 6:**
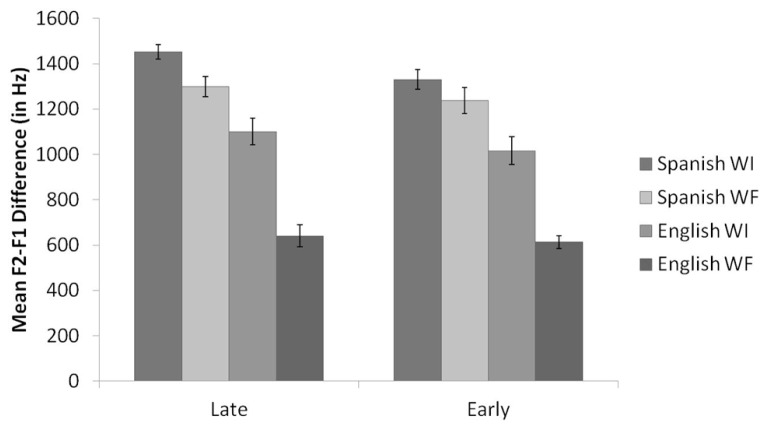
**Mean raw F2-F1 values by language and context for Late Bilinguals and Early Bilinguals.** Error bars refer to standard errors.

**Figure 6** shows the means and standard errors for the raw F2-F1 differences for Spanish and English /l/ productions once again, organized by language, background, and context. Results of the F2-F1 analyses revealed, once again, a significant difference across the four measures for Late Bilinguals, *F*(3,39) = 58.9, *p* < 0.001, and for Early Bilinguals, *F*(3,30) = 49.5, *p* < 0.001.

*Post hoc* tests using Bonferroni correction for F2-F1 values for the Late Bilinguals revealed that Spanish WI /l/s were significantly higher than Spanish WF, English WI, and English WF /l/s. In addition, Spanish WF /l/s were significantly higher than English WF /l/s, and English WI /l/s were significantly higher than English WF /l/s. (All *p*s < 0.02.) Spanish WF and English WI /l/s were not significantly different (*p* = 0.18).

*Post hoc* tests for the Early Bilinguals revealed that Spanish WI /l/s were significantly higher than English WI and WF /l/s. Moreover, English WI /l/s were higher than the English WF /l/s. (All *p*s < 0.01.) The difference between Spanish WF and English WI /l/s approached significance (*p* = 0.08), with Spanish WF /l/s higher than English WI /l/s, whereas Spanish WI and WF /l/s were not significantly different (*p* = 0.52).

These findings aid in interpretation of the results of the between-groups analysis for Spanish. The difference by context for Spanish /l/ productions is attributed to the productions of the Late Bilinguals. That is, the Late Bilinguals’ Spanish WI /l/s are clearer than their Spanish WF /l/s; in contrast, the Early Bilinguals’ Spanish /l/s do not differ significantly by context (though there is a trend in the same direction).

Taken together, these findings also show support for the prediction that the Bilinguals would produce a phonetic distinction between their English and Spanish productions; however, the two groups showed different profiles for these distinctions. Specifically, the Early Bilinguals produced both contextual /l/s in Spanish with higher normalized F2 and raw F2-F1 values than those in English. The Late Bilinguals, in contrast, only distinguished Spanish WI /l/s from the English /l/s. They did not produce a significant difference between their Spanish WF and English WI /l/s (though, once again, there was a trend in that direction).

**FIGURE 7:**
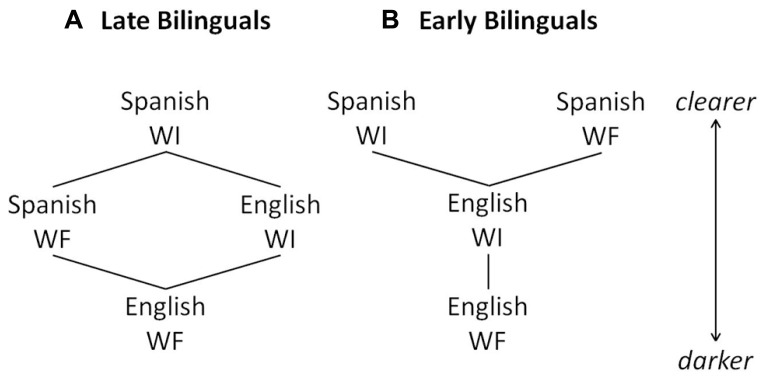
**Schematic of /l/ clearness by language and context for Late (A) and Early (B) Bilinguals**.

The results point to two distinct profiles for the Early and Late Bilingual groups, which are depicted in **Figure 7**, with **Figure 7A** representing Late Bilinguals and **Figure 7B** representing Early Bilinguals. Each /l/ variant by language and context is ranked according to clearness, based on the analyses of normalized F2 and raw F2-F1 values. Note that the profile represented by the Early Bilinguals (**Figure 7B**), is identical to comparisons of the two languages. That is, Spanish WI and WF /l/s show little to no difference in clearness, and are clearer than English WI /l/s, which in turn are clearer than English WF /l/s. The Late Bilingual profile (**Figure 7A**) thus differs from the monolingual pattern. Thus these combined results show us that the Early and Late Bilinguals both have knowledge of the phonological patterning of /l/ in English that is comparable to those of monolinguals. In Spanish, however, only the Early Bilinguals show monolingual-like knowledge of how /l/ patterns. The Late Bilinguals, in producing a difference by context, show a different type of phonological knowledge.

The combined results additionally support the prediction that Early and Late Bilinguals would differ from one another in their phonetic implementation of the Spanish and English /l/s. And though the two groups do differ from one another, they do not differ exactly as expected. Recall, it was predicted that the Late Bilinguals would show evidence of category assimilation, given that their Spanish phonetic categories were more established before acquisition of English began. The results do support this prediction, given that their English /l/s are clearer than those of the monolinguals, and their Spanish WF /l/s are not significantly different from their English WI /l/s. Yet, it was also predicted that the Early Bilinguals would show evidence of separate category formation due to the fact that Spanish phonetic categories were developing simultaneously (or nearly so) with English phonetic categories. Though the Early Bilinguals did distinguish their Spanish /l/s from their English /l/s, which is suggestive of category formation, their English /l/s were clearer than those of the monolinguals, which is suggestive of category assimilation, per Flege (1995).

## DISCUSSION

The goal of this study was to evaluate the phonetic and phonological characteristics of Spanish and English /l/ productions by two groups of bilinguals who differed in their AoA of English. The results are discussed in light of the hypotheses proposed in the Introduction.

It was predicted that Early and Late Bilinguals would show knowledge of the phonological systems of both English and Spanish. For English, they were expected to show knowledge of the allophonic velarization rule by producing darker /l/s in WF vs. WI position. Both the Early and Late Bilinguals showed this pattern of production in English, indicating that, despite differences in AoA of English, acquisition of the allophonic rule occurred. This pattern is consistent with English phonology, and was expected to occur given that even young bilingual children demonstrate knowledge of this allophonic pattern (Barlow et al., 2013). It was also not surprising given the high receptive and expressive abilities in English as reported by all participants in the study.

For Spanish, the bilinguals were expected to produce little to no difference by context for /l/ productions, since Spanish /l/s are similar across contexts. In fact, the Late Bilinguals produced darker /l/s in WF than in WI position. This would suggest that the Late Bilinguals show influence of the English phonological rule on their Spanish phonological system. That is, the English allophonic velarization rule has transferred into their Spanish phonological system. Unfortunately, there was not a Spanish monolingual group in the current study to further support this claim. (See below for further discussion of this limitation.) However, assuming that prior reports of the facts about Spanish /l/ are correct, it appears that knowledge of the English allophonic rule has influenced the Late Bilinguals’ productions of Spanish /l/.

It was also predicted that the bilinguals would produce a phonetic distinction between their Spanish and English /l/s, independent of the allophonic velarization pattern. This finding was generally supported: Spanish /l/s were clearer than English /l/s. However, the two groups differed from one another in terms of contextual variants. As depicted in **Figure 7**, the Early Bilinguals produced both Spanish WI and WF /l/s as clearer than both English WI and WF /l/s. This would suggest that the Early Bilinguals have formed separate phonetic categories for Spanish and English /l/s in each context, as was predicted, since they began to acquire the English phonetic categories before or at the same time as the Spanish phonetic categories were established (McLaughlin, 1978; Flege, 1991, 2007; Flege et al., 1995; Hamers and Blanc, 2000; Genesee et al., 2004; Lord, 2008; Gildersleeve-Neumann and Wright, 2010; Lee and Iverson, 2012). There was no evidence of category dissimilation for the Early Bilinguals, consistent with study predictions (Flege et al., 1995; Flege, 2002). That is, the Early Bilinguals’ Spanish /l/s were not clearer than those reported for Spanish monolinguals, nor were they clearer than those of the Late Bilinguals. Moreover, their English /l/s were not darker than those of the Monolinguals. In fact, their English WI /l/s were somewhat clearer than those of the Monolinguals, which would suggest category assimilation. Still, this difference was only marginally significant, so it is assumed that the Early Bilinguals did form separate phonetic categories for their Spanish and English /l/s.

For the Late Bilinguals, only Spanish WI /l/s were clearer than English /l/s. The Spanish WF /l/s did not differ from English WI /l/s (again, refer to **Figure 7**). Thus, the phonetic categories for the Spanish and English /l/s have not been formed in the same way as for the Early Bilinguals, since Spanish WF and English WI /l/s are not distinct. That is, their Spanish WF /l/s were produced as darker than Spanish WI /l/s (and darker than what is reported for monolingual Spanish WF /l/s in the literature), and their English WI /l/s were produced as clearer than those produced by Monolinguals. This is consistent with the prediction that the Late Bilinguals would show evidence of category assimilation by producing Spanish and English /l/s that were intermediate to those of monolinguals. Because the Late Bilinguals’ phonetic categories for Spanish were more established prior to acquisition of English (Flege, 1991; Flege et al., 1995; Lee and Iverson, 2012), they may have been less likely to establish separate phonetic categories for the English and Spanish /l/ phones, and therefore merged them, leading to some /l/ productions that were not distinct.

Taking the results together, these show that there is a bidirectional influence between a bilingual’s two languages, as predicted by the SLM (Flege, 1995), but this only seems apparent for the Late Bilinguals. That is, it appears that the Early Bilinguals show a slight Spanish-to-English *phonetic* influence, making the /l/s clearer. The Late Bilinguals also show this Spanish-to-English phonetic influence, and to a greater degree; however, they also show an English-to-Spanish *phonological* influence, given that the English allophonic rule also applies in their Spanish productions.

Documentation of L2-to-L1 interactions has been of particular focus in recent research on bilinguals and L2 learners (Cook, 2003; Kecskes, 2008). Such interaction has been reported for both children and adults and for a variety of linguistic structures, including but not limited to use of narrative structures (Pavlenko and Jarvis, 2002), processing relative clauses (Dussias and Sagarra, 2007), the use of PRO-drop (Satterfield, 2003), intonational patterns (Mennen, 2004), stress (Paradis, 2001a, b), vowel formants (Chang, 2012), and of course VOT (Flege, 1987; Thornburgh and Ryalls, 1998; Riney and Okamura, 1999; Whitworth, 2000; Zampini and Green, 2001; Kehoe et al., 2004; Lord, 2008; López, 2012).

Thus, the finding that the L2 (in this case, English) influences the L1 (Spanish) is not novel; however, the findings presented herein add to the body of research on L2-to-L1 influences, particularly for adults who acquired their L2 prior to adulthood. Interestingly, this influence was only apparent in the Late Bilinguals, and specifically in terms of the *phonological* system. Granted, the Early Bilinguals showed a similar, though non-significant, trend in the same direction. Possibly, with greater numbers of speakers a similar pattern would have been observed for that group as well.

The fact that a phonological pattern transferred from the Late Bilinguals’ L2 to the L1 is surprising, given the proposed cutoff age of 5 years for the critical period for phonology, as discussed in the Introduction (Flege et al., 1999; Scovel, 2000; Newport et al., 2001). Recall, effects of the L1 on the L2 are greater after this age; prior research has not implicated the effects of the L2 on the L1. In this particular case, a new phonological rule of the L2 was not only acquired, it also impacted the L1. This suggests that both phonetic and phonological learning (and change) can continue past the age of 5 years, and can impact the L1. Nevertheless, the Late Bilinguals showed a greater degree of interaction between their two languages than did the Early Bilinguals, which suggests that the difference in AoA for English was a distinguishing factor. Perhaps acquiring a L2 after the critical period for phonology makes the learner less able to accommodate “competing” phonological patterns of their two languages. Acquisition of a phonological rule in the L2 was successful, but came at a cost to the L1.

The novel contribution of this study is that it focuses not only on the phonetic differences between bilinguals’ productions of speech sounds that are shared between their two languages, but also on the knowledge and application of a phonological rule that affects those sounds in a particular context. Future studies that evaluate bilinguals’ acquisition and use of an allophonic pattern should consider not only the allophones in those contexts that are affected by the allophonic rule (as with syllable-final /l/ for English), but also those contexts where the rule does not apply (as with syllable-initial /l/), because bilinguals may exhibit interaction between their two languages in those contexts as well, either in terms of category assimilation or, perhaps, dissimilation.

For instance, future research should evaluate bilinguals’ productions of other allophonic patterns for phonemes that are shared between their two languages. Consider the comparison of VOT measures of voiceless oral stops in English and Spanish. As mentioned above, VOT in Spanish-English bilinguals is often studied because English has an allophonic rule that governs the distribution of long- and short-lag voiceless stops, whereas Spanish has only short-lag voiceless stops. It would be important to evaluate the English allophones in both long- and short-lag contexts, and to also evaluate Spanish stops in those same contexts. Given the findings from the present study, we might predict that both Early and Late Bilinguals would show a Spanish-to-English phonetic influence, by causing the English stops to have shorter lags. In addition, we might also predict that the Late Bilinguals would show an English-to-Spanish phonetic influence, such that the rule of aspiration also affects their Spanish stops, making them longer in the same context as in English.

Moreover, such studies should also take into consideration whether the allophonic pattern in question is part of the L1 or the L2 in the case of late bilinguals. For instance, an evaluation of /l/ contextual phones as produced by English-Spanish bilinguals (whose L1 is English) would be of particular interest, given the findings of the current study. We might still predict that the early English-Spanish bilinguals would show influence of English on Spanish. However, for English-Spanish late bilinguals, evaluating their suppression of the English /l/-velarization pattern in Spanish would be of particular interest. Perhaps their use of the allophonic rule would be diminished in English, due to influence of Spanish. Or perhaps the velarization pattern would extend to their Spanish productions, just as with the Late Bilinguals in the current study.

An obvious limitation to the current study is the absence of data from Spanish monolinguals. Such information would have provided additional support for the claim that the bilinguals’ Spanish /l/ productions were influenced by their knowledge of English. Finding such participants would be a challenge, at least for this particular region of the US and Mexico. Recall that all participants in the current study were college students. It would be difficult, if not impossible, to find college students in Baja California (let alone Southern California) regions who are monolingual Spanish speakers, because English language classes are common in many private and some public school curricula in Mexico, and are compulsory at the university level (Sierra and Padilla, 2003; O’Donnell, 2010; Torres-Olave, 2012). Of course, similar-aged monolingual Spanish participants who do not attend university could have been included in the current study, but their inclusion might have introduced production patterns associated with sociolinguistic factors other than their monolingual status (Lippi-Green, 1997; Lipski, 2008; Coloma, 2011). Future studies should include a larger and more diverse group of adult Spanish- and/or English-speakers to allow for an in-depth comparison of Spanish /l/ as spoken by Spanish-English bilinguals vs. Spanish monolinguals in order to determine the extent to which knowledge of English influences pronunciation of Spanish /l/. Moreover, it may be necessary to further evaluate Spanish and English regional dialect features. Perhaps phonetic and phonological characteristics of Spanish are in the process of changing due to contact with English and vice versa (Goebl et al., 1996). Consider that Chicano English is characterized as having a clearer /l/ than other dialects of English, though this varies across generations (Frazer, 1996; Van Hofwegen, 2009). This too presents a challenge, because it would be difficult to tease apart effects of a given Spanish-Chicano English bilingual speaker’s knowledge of Spanish from the effects of the Chicano English dialect, since the dialect has numerous properties that are attributed to Spanish influence (Fought, 2003).

It may be that differences between the Early and Late Bilinguals are attributable to greater variability in Spanish proficiency for the former group and in English proficiency for the latter group. Despite the balanced ratings for expressive and receptive abilities and for input and output across the groups, it is possible that Early Bilinguals were more balanced bilinguals because of their earlier acquisition of English, and were also more homogeneous in terms of their abilities in their two languages as compared to the Late Bilinguals. It is well-documented that the later a language is acquired, the more variability there will be in the extent to which that language is acquired (Birdsong, 2006). Indeed, the Late Bilinguals did have a larger English AoA range, as evidenced by a larger AoA standard deviation.

Future studies should also compare Spanish-English bilinguals who learned Spanish first with those who learned English first, and should also compare differences in language dominance, given that dominance can change over time and is not necessarily dependent on which language was acquired first. As stated previously, language dominance is a critical factor in determining the direction of influence between the L1 and L2 (Flege and Eefting, 1987; Flege, 1991; Flege et al., 1995, 2002; Simonet, 2010; Antoniou et al., 2011). Generally speaking, early bilinguals tend to be dominant in the L2, whereas late bilinguals tend to remain dominant in the L1 (Flege et al., 2002). It is difficult to determine what the L1 and the L2 are in the case of the Early Bilinguals in the present study, given that the children were exposed to both languages from a very young age. Moreover, both groups appeared to be dominant in English based on their input and output scores, yet both also showed a phonetic influence of Spanish on their English productions. The direction of this influence would implicate Spanish as the dominant language for *both* groups, despite their input and output scores on the questionnaire, which may not have been sensitive enough. Otherwise, we might have expected the reverse pattern of phonetic influence, at least for the Early Bilinguals. On the other hand, the Late Bilinguals showed the unexpected impact of English (their L2) on the Spanish phonological system; this is suggestive of English dominance.

Though an attempt was made to match the Early and Late Bilinguals in terms of input, output, and proficiency, the language use questionnaire employed for this study did not document the extent to which code-switching was employed by the two groups, and this too could have impacted the results. Perhaps the Early Bilinguals code-switched more regularly, and may have done so since early childhood. The Late Bilinguals may have code-switched less at the time of the study, and also during the process of acquiring English. Though there is conflicting evidence regarding the cross-language phonetic and phonological influence of one language on the other during code-switched utterances (Grosjean and Miller, 1994; Bullock et al., 2004), the current study attempted to prevent opportunities to code-switch by separating the tasks in the two languages and requiring the participants to read the word lists in their carrier phrases. However, the long-term impact of different levels of code-switching that may have distinguished the two groups was not controlled for (Balukas and Koops, in press). Perhaps regular code-switching is more likely to lead to category assimilation, whereas less frequent code-switching might serve to maintain separate phonetic categories. This too would be an interesting direction for future research.

In summary, the results of the foregoing study indicate that AoA does impact bilinguals’ production of sounds that are shared between their two languages. Although there was a *phonetic* influence from Spanish to English regardless of the age at which English was acquired, this was stronger for those bilinguals who learned English at a later age. Moreover, the effects of English *phonology* on Spanish were only apparent for those bilinguals who acquired English at a *later* age. The findings raise a number of questions regarding AoA, dominance, and the direction of influence that would be fruitful directions for continued study of bilingual phonology.

## Conflict of Interest Statement

The author declares that the research was conducted in the absence of any commercial or financial relationships that could be construed as a potential conflict of interest.

## Footnotes

1 Note that English dialects vary by /l/ darkness. American English /l/ is described as dark in all contexts; however, many (but not all) British dialects employ a clear /l/ in onset position and a dark /l/ only in the rhyme (Hawkins and Nguyen, 2004; Carter and Local, 2007). Moreover, interspeaker variability in /l/ darkness has also been observed for American English (Huffman, 1997).

2 For expository purposes, the term “Late Bilingual” is used. These bilinguals are often referred to as “sequential bilinguals” or “heritage speakers” (Valdés, 2005). The term “sequential” is intentionally avoided here, because some of the Early Bilinguals are arguably characterized as sequential as well, having learned English later than Spanish (e.g., at age 3 years; Meisel, 2001; Genesee et al., 2004).

3 The standard errors for the monolinguals reflect a common situation in a bilingual context. Spanish is prevalent in this community and can be heard in marketplaces, in restaurants, and on television and radio.

4 Intervocalic forms were excluded because English and Spanish differ in terms of how intervocalic /l/ is syllabified, particularly in post-tonic contexts. Thus, in the English word “solo,” the /l/ is often assumed to occur in coda position of the first syllable, as with [ˈsoɫ.o], due to stress-based (re-)syllabification, though lexical and morphological factors also play a role (Borowsky, 1986; Hayes, 2000; Yuan and Liberman, 2011; Lee-Kim et al., 2013). Whereas, in the Spanish word *solo*, the /l/ is assumed to occur in onset position, as [ˈso.lo] (Harris, 1983; Colina, 1997). This is an “interlanguage structural ambiguity” (Paradis, 2001b) that would be of interest for future studies on Spanish-English bilinguals. Plosive + /l/ forms were also excluded due to inherent differences in VOT that occur on the so-called voiced and voiceless plosives, which in turn can affect characteristics and subsequent identification of the formants for the following /l/ (Flege and Eefting, 1987; Flege, 1991; Yavaş, 2008).

5 An earlier draft of this paper included a normalization process that used the /i/ and /u/ vowels. Given the fronter quality of California English /u/, a normalization process using the vowels /i/ and /o/ was recommended as an alternative by an anonymous reviewer. The results of this prior analysis were similar to the findings presented here, which strengthens the findings observed here.

## References

[B1] Adank P. Smits R van Hout R. (2004). A comparison of vowel normalization procedures for language variation research. *J. Acoust. Soc. Am.* 116 3099–3107 10.1121/1.1795335 15603155

[B2] Amengual M. (2012). Interlingual influence in bilingual speech: cognate status effect in a continuum of bilingualism. *Biling. Lang. Cogn.* 15 517–530 10.1017/S1366728911000460

[B3] Antoniou M. Best C. T. Tyler M. D. Kroos C. (2011). Inter-language interference in VOT production by L2-dominant bilinguals: asymmetries in phonetic code-switching. *J. Phon.* 39 558–570 10.1016/j.wocn.2011.03.001 22787285 PMC3391600

[B4] Balukas C. Koops C. (in press). Spanish-English bilingual voice onset time in spontaneous code-switching. *Int. J. Biling.* 10.1177/1367006913516035

[B5] Barlow J. A. (2003). The stop-spirant alternation in Spanish: converging evidence for a fortition account. *Southwest J. Linguist.* 22 51–86

[B6] Barlow J. A. Branson P. E Nip I. S. B. (2013). Phonetic equivalence in the acquisition of /l/ by Spanish-English bilingual children. *Biling. Lang. Cogn.* 16 68–85 10.1017/S1366728912000235

[B7] Birdsong D. (2006). Age and second language acquisition and processing: a selective overview. *Lang. Learn.* 56 9–49 10.1111/j.1467-9922.2006.00353.x

[B8] Boersma P. Weenink D. (2008). *Praat: Doing Phonetics by Computer* v. 5.0.26. Retrieved June 16, 2008, from http://www.praat.org

[B9] Borowsky T. (1986). *Topics in the Lexical Phonology of English*. Doctoral dissertation, University of Massachusetts Amherst

[B10] Broselow E. (2004). Unmarked structures and emergent rankings in second language phonology. *Int. J. Biling.* 8 51–65 10.1177/13670069040080010401

[B11] Browman C. Goldstein L. (1992). Articulatory phonology: an overview. *Phonetica* 49 155–180 10.1159/000261913 1488456

[B12] Bullock B. E. Toribio A. J. Davis K. A. Botero C. G. (2004). “Phonetic convergence in bilingual Puerto Rican Spanish,” in *WCCFL 23: Proceedings of the 23rd West Coast Conference on Formal Linguistics* eds Chand V. Kelleher A. Rodríguez A. J. Schmeiser B. (Somerville, MA: Cascadilla Proceedings Project) 113–125

[B13] Carter P. Local J. (2007). F2 variation in Newcastle and Leeds English liquid systems. *J. Int. Phon. Assoc.* 37 183–199 10.1017/S0025100307002939

[B14] Chang C. B. (2012). Rapid and multifaceted effects of second-language learning on first-language speech production. *J. Phon.* 40 249–268 10.1016/j.wocn.2011.10.007

[B15] Chládková K. Escudero P. Boersma P. (2011). Context-specific acoustic differences between Peruvian and Iberian Spanish vowels. *J. Acoust. Soc. Am.* 130 416–428 10.1121/1.3592242 21786909

[B16] Colina S. (1997). Identity constraints and Spanish resyllabification. *Lingua* 103 1–23 10.1016/S0024-3841(97)00011-9

[B17] Coloma G. (2011). Valoración socioeconómica de los rasgos fonéticos dialectales de la lengua española [Socio-economic assessment of dialectal phonetic features of the Spanish language]. *Lexis* 35 91–118

[B18] Cook V. (ed.) (2003). *Effects of the Second Language on the First*. Buffalo, NY: Multilingual Matters Ltd

[B19] Dussias P. E. (2003). Syntactic ambiguity resolution in L2 learners: some effects of bilinguality on L1 and L2 processing strategies. *Stud. Second Lang. Acquis.* 25 529–557 10.1017/S0272263103000238

[B20] Dussias P. E. Sagarra N. (2007). The effect of exposure on syntactic parsing in Spanish-English bilinguals. *Biling. Lang. Cogn.* 10 101–116 10.1017/S1366728906002847

[B21] Eckman F. R. Iverson G. K. (1997). “Structure preservation in interlanguage phonology,” in *Focus on Phonological Acquisition* eds Hannahs S. J. Young-Scholten M. (Philadelphia: John Benjamins) 183–208

[B22] Fabiano L. C. Goldstein B. A. (2005). Phonological cross-linguistic effects in bilingual Spanish-English speaking children. *J. Multiling. Commun. Disord.* 3 56–63 10.1080/14769670400027316

[B23] Fabiano-Smith L. Barlow J. A. (2010). Interaction in bilingual phonological acquisition: evidence from phonetic inventories. *Int. J. Biling. Educ. Biling.* 13 81–97 10.1080/13670050902783528 20126516 PMC2802227

[B24] Fabiano-Smith L. Goldstein B. A. (2010). Phonological acquisition in bilingual Spanish-English speaking children. *J. Speech Lang. Hear. Res.* 53 160–178 10.1044/1092-4388(2009/07-0064) 20150407

[B25] Flege J. Frieda E. Nozawa T. (1997). Amount of native language (L1) use affects the pronunciation of an L2. *J. Phon.* 25 160–186 10.1006/jpho.1996.0040

[B26] Flege J. MacKay I. (2004). Perceiving vowels in a second language. *Stud. Second Lang. Acquis.* 26 1–34 10.1017/S0272263104026117

[B27] Flege J. E. (1987). The production of ‘new’ and ‘similar’ phones in a foreign language: evidence for the effect of equivalence classification. *J. Phon.* 15 47–65

[B28] Flege J. E. (1991). Age of learning affects the authenticity of voice onset time (VOT) in stop consonants produced in a second language. *J. Acoust. Soc. Am.* 89 395–411 10.1121/1.400473 2002177

[B29] Flege J. E. (1995). “Second language speech learning: theory, findings and problems,” in *Speech Perception and Linguistic Experience: Theoretical and Methodological Issues* ed. Strange W. (Baltimore: York Press) 233–277

[B30] Flege J. E. (2002). “Interactions between native and second-language phonetic subsystems,” in *An Integrated View of Language Development: Papers in Honor of Henning Wode* eds Burmeister P. Piske T. Rohde A. (Trier: Wissenschaftlicher Verlag) 217–244

[B31] Flege J. E. (2007). “Language contact in bilingualism: phonetic system interactions,” in *Laboratory Phonology* Vol. 9 eds Cole J. Hualde J. I. (Berlin: Mouton de Gruyter) 353–382

[B32] Flege J. E. Eefting W. (1987). Production and perception of English stops by native Spanish speakers. *J. Phon.* 15 67–83

[B33] Flege J. E. MacKay I. (2011). “What accounts for “age” effects on overall degree of foreign accent?,” in *Achievements and Perspectives in the Acquisition of Second Language Speech: New Sounds 2010* eds Wrembel M. Kul M. Dziubalska-Kołaczyk K. (Bern: Peter Lang) 65–82

[B34] Flege J. E. MacKay I. R. A. Piske T. (2002). Assessing bilingual dominance. *Appl. Psycholinguist.* 23 567–598 10.1017/S0142716402004046

[B35] Flege J. E. Munro M. J MacKay I. R. A. (1995). Effects of age of second-language learning on the production of English consonants. *Speech Commun.* 16 1–26 10.1016/0167-6393(94)00044-B

[B36] Flege J. E. Schirru C MacKay I. R. A. (2003). Interaction between the native and second language phonetic subsystems. *Speech Commun.* 40 467–491 10.1016/S0167-6393(02)00128-0

[B37] Flege J. E. Yeni-Komshian G. H. Liu S. (1999). Age constraints on second-language acquisition. *J. Mem. Lang.* 41 78–104 10.1006/jmla.1999.2638

[B38] Flynn N. Foulkes P. (2011). “Comparing vowel formant normalization methods,” in *Proceedings of the 17th International Congress of Phonetic Sciences (ICPhS XVII)* eds Lee W.-S. Zee E. (Hong Kong: City University of Hong Kong) 683–686

[B39] Fought C. (2003). *Chicano English in Context*. New York: Macmillan

[B40] Fowler C. A. Sramko V. Ostry D. J. Rowland S. A Hallé P. (2008). Cross language phonetic influences on the speech of French–English bilinguals. *J. Phon.* 36 649–663 10.1016/j.wocn.2008.04.001 19802325 PMC2598425

[B41] Frazer T. C. (1996). Chicano English and Spanish interference in the Midwestern United States. *Am. Speech* 71 72–85 10.2307/455470

[B42] Genesee F. Paradis J. Crago M. B. (2004). *Dual Language Development and Disorders: A Handbook on Bilingualism and Second Language Learning*. Baltimore: Brookes

[B43] Gick B. (2003). “Articulatory correlates of ambisyllabicity in English glides and liquids,” in *Phonetic Interpretation (Papers in Laboratory Phonology VI)* eds Local J. Ogden R. Temple R. (Cambridge: Cambridge University Press) 222–236

[B44] Gick B. Campbell F. Oh S. Tamburri-Watt L. (2006). Toward universals in the gestural organization of syllables: a cross-linguistic study of liquids. *J. Phon.* 34 49–72 10.1016/j.wocn.2005.03.005

[B45] Gick B. W. (2000). *The Articulatory Basis of Syllable Structure: A Study of English Glides and Liquids*. Doctoral dissertation, Yale University New Haven

[B46] Gildersleeve-Neumann C. E. Wright K. L. (2010). English speech acquisition in 3- to 5-year-old children learning Russian and English. *Lang. Speech Hear. Serv. Sch.* 41 429–444 10.1044/0161-1461(2009/09-0059) 20421617

[B47] Goebl H. Nelde P. H. Stary Z. Woelck W. (1996). *Kontaktlinguistik/Contact Linguistics/Linguistique de Contact: Ein Internationales Handbuch Zeitgenoessischer Forschung/An International Handbook of Contemporary Research/Manuel International Des Recherches Contemporaines*. New York: Walter de Gruyter

[B48] Grijalva C. Piccinini P. E. Arvaniti A. (2013). The vowel spaces of Southern Californian English and Mexican Spanish as produced by monolinguals and bilinguals. *J. Acoust. Soc. Am.* 33 3340 10.1121/1.4800752

[B49] Grosjean F. Miller J. L. (1994). Going in and out of languages: an example of bilingual flexibility. *Psychol. Sci.* 5 201–206 10.1111/j.1467-9280.1994.tb00501.x

[B50] Guion S. G. (2003). The vowel systems of Quichua-Spanish bilinguals: age of acquisition effects on the mutual influence of the first and second languages. *Phonetica* 60 98–128 10.1159/000071449 12853715

[B51] Gutiérrez-Clellen V. F. Kreiter J. (2003). Understanding child bilingual acquisition using parent and teacher reports. *Appl. Psycholinguist.* 24 267–288 10.1017/S0142716403000158

[B52] Hagiwara R. (1995). *Acoustic Realizations of American /r/ as Produced by Women and Men*. Doctoral dissertation, University of California Los Angeles

[B53] Hagiwara R. (1997). Dialect variation and formant frequency: the American English vowels revisited. *J. Acoust. Soc. Am.* 102 655–658 10.1121/1.419712

[B54] Hamers J. F Blanc M. H. A. (2000). *Bilinguality and Bilingualism*. Cambridge: Cambridge University Press 10.1017/CBO9780511605796

[B55] Harris J. W. (1983). *Syllable Structure and Stress in Spanish*. Cambridge, MA: MIT Press

[B56] Hawkins S. Nguyen N. L. (2004). Influence of syllable-coda voicing on the acoustic properties of syllable-onset /l/ in English. *J. Phon.* 32 199–231 10.1016/S0095-4470(03)00031-7

[B57] Hayes B. P. (2000). “Gradient well-formedness in optimality theory,” in *Optimality Theory: Phonology, Syntax, and Acquisition* eds Dekkers J. Van Der Leeuw F. Van De Weijer J. (New York: Oxford) 88–120

[B58] Hazan V. Barrett S. (2000). The development of phonemic categorization in children aged 6–12. *J. Phon.* 28 377–396 10.1006/jpho.2000.0121

[B59] Hillenbrand J. Getty L. A. Clark M. J. Wheeler K. (1995). Acoustic characteristics of American English vowels. *J. Acoust. Soc. Am.* 97 3099–3111 10.1121/1.411872 7759650

[B60] Hualde J. I. (2005). *The Sounds of Spanish*. New York: Cambridge University Press

[B61] Huffman M. K. (1997). Phonetic variation in intervocalic onset /l/’s in English. *J. Phon.* 25 115–141 10.1006/jpho.1996.0038

[B62] Kecskes I. (2008). The effect of the second language on the first language. *Babylonia* 2 30–34

[B63] Kehoe M. M. Lleó C. Rakow M. (2004). Voice onset time in bilingual German-Spanish children. *Biling. Lang. Cogn.* 7 71–88 10.1017/S1366728904001282

[B64] Kent R. D. (1976). Anatomical and neuromuscular maturation of the speech mechanism: evidence from acoustic studies. *J. Speech Hear. Res.* 19 421–447 10.1044/jshr.1903.421 979206

[B65] Labov W. Ash S. Boberg C. (2006). *Atlas of North American English: Phonetics, Phonology, and Sound Change*. New York: Mouton de Gruyter

[B66] Ladefoged P. Johnson K. (2011). *A Course in Phonetics*. Boston, MA: Cengage Learning

[B67] Lee S. A. S. Iverson G. K. (2012). Stop consonant productions of Korean–English bilingual children. *Biling. Lang. Cogn.* 15 275–287 10.1017/S1366728911000083

[B68] Lee-Kim S.-I. Davidson L. Hwang S. (2013). Morphological effects on the darkness of English intervocalic /l/. *Lab. Phonol.* 4 475–511 10.1515/lp-2013-0015

[B69] Lehiste I. (1964). *Some Acoustic Characteristics of Selected English Consonants*. Bloomington, IN: Indiana University Research Center in Anthropology, Folklore, and Linguistics

[B70] Lippi-Green R. (1997). *English with an Accent: Language, Ideology, and Discrimination in the United States*. New York: Routledge

[B71] Lipski J. M. (2008). Varieties of Spanish in the United States. Washington, DC: Georgetown University Press

[B72] Lleó C. (2006). The acquisition of prosodic word structure in Spanish by monolingual and Spanish-German bilingual children. *Lang. Speech* 49 205-229 10.1177/00238309060490020401 17037122

[B73] Lleó C. Kuchenbrandt M. Kehoe M. Trujillo C. (2003). “Syllable final consonants in Spanish and German monolingual and bilingual acquisition,” in *(In)vulnerable Domains in Multilingualism* ed. Müller N. (Philadelphia: John Benjamins) 191–220

[B74] López V. G. (2012). Spanish and English word-initial voiceless stop production in code-switched vs. monolingual structures. *Second Lang. Res.* 28 243–263 10.1177/0267658312439821

[B75] Lord G. (2008). “Second language acquisition and first language phonological modification,” in *Selected Proceedings of the 10th Hispanic Linguistics Symposium* eds Bruhn De Garavito J. Valenzuela E. (Somerville, MA: Cascadilla Proceedings Project) 184–193

[B76] MacKay I. Flege J. Piske T. Schirru C. (2001). Category restructuring during second-language (L2) speech acquisition. *J. Acoust. Soc. Am.* 110 516–528 10.1121/1.1377287 11508976

[B77] MacLeod A. A. N. Stoel-Gammon C. (2010). What is the impact of age of second language acquisition on the production of consonants and vowels among childhood bilinguals? *Int. J. Biling.* 14 400–421 10.1177/1367006910370918

[B78] MacWhinney B. (2004). “A unified model of language acquisition,” in *Handbook of Bilingualism: Psycholinguistic Approaches* eds Kroll J. De Groot A. (New York: Oxford University Press) 49–67

[B79] McLaughlin B. (1978). *Second-Language Acquisition in Childhood*. Mahwah, NJ: Lawrence Erlbaum Associates

[B80] Meisel J. M. (2001). “The simultaneous acquisition of two languages: early differentiation and subsequent development of grammars,” in *Trends in Bilingual Acquisition* eds Cenoz J. Genesee F. (Philadelphia: John Benjamins) 11–42

[B81] Mennen I. (2004). Bi-directional interference in the intonation of Dutch speakers of Greek. *J. Phon.* 32 543–563 10.1016/j.wocn.2004.02.002

[B82] Newport E. L. Bavelier D. Neville H. J. (2001). “Critical thinking about critical periods: perspectives on a critical period for language acquisition,” in *Language, Brain and Cognitive Development: Essays in Honor of Jaques Mehler* ed. Dupoux E. (Cambridge, MA: MIT Press) 481–502

[B83] O’Donnell J. L. (2010). The indigenous, national, and international language in higher education: students’ academic trajectories in Oaxaca, Mexico. *Int. J. Appl. Linguist.* 20 386–416 10.1111/j.1473-4192.2010.00254.x

[B84] Oh S. (2005). *Articulatory Characteristics of English /l/ in Speech Development*. Doctoral dissertation, University of British Columbia Vancouver

[B85] Oxley J. Roussel N. Buckingham H. W. (2007). Contextual variability in American English dark-l. *Clin. Linguist. Phon.* 21 523–542 10.1080/02699200701356485 17564855

[B86] Paradis J. (2001a). “Beyond ‘one system or two?’: degrees of separation between the languages of French–English bilingual children,” in *Cross-Linguistic Structures in Simultaneous Bilingualism* ed. Döpke S. (Amsterdam: John Benjamins) 175–200

[B87] Paradis J. (2001b). Do bilingual two-year-olds have separate phonological systems? *Int. J. Biling.* 5 19–38 10.1177/13670069010050010201

[B88] Pavlenko A. Jarvis S. (2002). Bidirectional transfer. *Appl. Linguist.* 23 190–214 10.1093/applin/23.2.190

[B89] Peterson G. E. Barney H. L. (1952). Control methods used in a study of the identification of vowels. *J. Acoust. Soc. Am.* 24 175–185 10.1121/1.1906875

[B90] Port R. F. Mitleb F. M. (1983). Segmental features and implementation of English by Arabic speakers. *J. Phon.* 11 219–229

[B91] Proctor M. (2010). *Gestural Characterization of a Phonological Class: The Liquids*. Doctoral dissertation, Yale University New Haven

[B92] Quilis A. Esgueva M. Gutiérrez Araus M. L. Cantarero M. (1979). Características acústicas de las consonantes laterales españoles. *Lingüíst. Esp. Actual.* 1 233–343

[B93] Recasens D. (2004). Darkness in [l] as a scalar phonetic property: implications for phonology and articulatory control. *Clin. Linguist. Phon.* 18 593–603 10.1080/02699200410001703556 15573493

[B94] Recasens D. (2012). A cross-language acoustic study of initial and final allophones of /l/. *Speech Commun.* 54 368–383 10.1016/j.specom.2011.10.001

[B95] Recasens D. Espinosa A. (2005). Articulatory, positional and coarticulatory characteristics for clear /l/ and dark /l/: evidence from two Catalan dialects. *J. Int. Phon. Assoc.* 35 1–25 10.1017/S0025100305001878

[B96] Riney T. J. Okamura K. (1999). Does bilingualism affect the first language? ICU Lang. *Res. Bull.* 14 101–113

[B97] Satterfield T. (2003). “Economy of interpretation: patterns of pronoun selection in transitional bilinguals,” in *Effects of the Second Language on the First* ed. Cook V. (Buffalo, NY: Multilingual Matters Ltd) 214–233

[B98] Scovel T. (2000). A critical review of the critical period research. *Annu. Rev. Appl. Linguist.* 20 213–223 10.1017/S0267190500200135

[B99] Shriberg L. Austin D. Lewis B. A. McSweeny J. Wilson D. (1997). The speech disorders classification systems (SDCS): extensions and lifespan reference data. *J. Speech Lang. Hear. Res.* 40 723–740 9263939 10.1044/jslhr.4004.723

[B100] Sierra A. M. Padilla A. (2003). “United States’ hegemony and purposes for learning English in Mexico,” in *Language: Issues of Inequality* eds Ryan P. M. Terborg R. (Mexico, DF: Universidad Nacional Autónoma de México) 215–232

[B101] Simonet M. (2010). Dark and clear laterals in Catalan and Spanish: interaction of phonetic categories in early bilinguals. *J. Phon.* 38 663–678 10.1016/j.wocn.2010.10.002

[B102] Sproat R. Fujimura O. (1993). Allophonic variation in English /l/ and its implications for phonetic implementation. *J. Phon.* 21 291–311

[B103] Thornburgh D. F. Ryalls J. H. (1998). Voice onset time in Spanish-English bilinguals: early versus late learners of English. *J. Commun. Disord.* 31 215–229 10.1016/S0021-9924(97)00053-1 9621904

[B104] Torres-Olave B. M. (2012). Imaginative geographies: identity, difference, and English as the language of instruction in a Mexican university program. *High. Educ.* 63 317–335 10.1007/s10734-011-9443-x

[B105] US Census Bureau. (2003). *Table 1. Language Use, English Ability, and Linguistic Isolation for the Population 5 Years and Over by State: 2000* [Online]. Available at: http://www.census.gov/population/cen2000/phc-t20/tab01.pdf [accessed on September 12, 2004]

[B106] US Census Bureau. (2004). *Table 6a. California – Ability to Speak English by Language Spoken at Home for the Population 5 Years and Over: 2000* [Online]. Available at: http://www.census.gov/population/cen2000/phc-t37/tab06a.pdf [accessed September 12, 2004]

[B107] Valdés G. (2005). Bilingualism, heritage language learners, and SLA research: opportunities lost or seized? *Mod. Lang. J.* 89 410–426 10.1111/j.1540-4781.2005.00314.x

[B108] Van Hofwegen J. (2009). Cross-generational change in /l/ in Chicano English. *English World Wide* 30 302–325 10.1075/eww.30.3.04van

[B109] Vorperian H. K. Wang S. Chung M. K. Schimek E. M. Durtschi R. B. Kent R. D. (2009). Anatomic development of the oral and pharyngeal portions of the vocal tract: an imaging study. *J. Acoust. Soc. Am.* 125 1666–1678 10.1121/1.3075589 19275324 PMC2669667

[B110] Walsh B. Smith A. (2002). Articulatory movements in adolescents: evidence for protracted development of speech motor control processes. *J. Speech Lang. Hear. Res.* 45 1119–1133 10.1044/1092-4388(2002/090) 12546482

[B111] Watt D. Fabricius A. (2002). Evaluation of a technique for improving the mapping of multiple speakers’ vowel spaces in the F1–F2 plane. *Leeds Work. Pap. Linguist.* 9 159–163

[B112] Wells J. C. (1982a). *Accents of English 1: An Introduction*. Cambridge: Cambridge University Press

[B113] Wells J. C. (1982b). *Accents of English 2: The British Isles*. Cambridge: Cambridge University Press

[B114] Whitley M. S. (2002). *Spanish/English Contrasts*. Washington, DC: Georgetown University Press.

[B115] Whitworth N. (2000). Acquisition of VOT and vowel length by English–German bilinguals: a pilot study. *Leeds Work. Pap. Linguist. Phon.* 8 229–243

[B116] Yavaş M. (2008). “Factors influencing the VOT of English long lag stops and interlanguage phonology,” in *New Sounds 2007: Proceedings of the Fifth International Symposium on the Acquisition of Second Language Speech* eds Rauber A. S. Watkins M. A. Baptista B. O. (Florianópolis: Federal University of Santa Catarina) 492–498

[B117] Yavaş M. S. (1996). “Differences in voice onset time in early and later Spanish-English bilinguals,” in *Spanish in Contact: Issues in Bilingualism* eds Roca A. Jensen J. B. (Somerville, MA: Cascadilla Press) 131–141

[B118] Yuan J. Liberman M. (2009). “Investigating /l/ variation in English through forced alignment,” in *Proceedings of Interspeech 2009* Brighton 2215–2218

[B119] Yuan J. Liberman M. (2011). /l/ variation in American English: a corpus approach. *J. Speech Sci.* 1 35–46

[B120] Zampini M. L. (1994). The role of native language transfer and task formality in the acquisition of Spanish spirantization. *Hispania* 77 470–481 10.2307/344974

[B121] Zampini M. L. (1996). Voiced stop spirantization in the ESL speech of native speakers of Spanish. *Appl. Psycholinguist.* 17 335–354 10.1017/S0142716400007979

[B122] Zampini M. L. Green K. P. (2001). “The voicing contrast in English and Spanish: the relationship between perception and production,” in *One Mind, Two Languages* ed. Nicol J. L. (Boston: Blackwell) 23–48

